# A phase II study of concurrent chemoradiotherapy with 5-fluorouracil and mitomycin-C for squamous cell carcinoma of the anal canal (the JROSG 10–2 trial)

**DOI:** 10.1093/jrr/rrac069

**Published:** 2022-10-23

**Authors:** Keiko Nemoto Murofushi, Satoshi Itasaka, Mototsugu Shimokawa, Yuji Murakami, Takaya Yamamoto, Yasumasa Nishimura, Shigehiro Kudo, Takashi Sakamoto, Takuro Ariga, Etsuyo Ogo, Kentaro Taguchi, Keiichi Jingu, Kazuhiko Ogawa

**Affiliations:** Division of Radiation Oncology, Department of Radiology, Tokyo Metropolitan Cancer and Infectious Diseases Center Komagome Hospital, Tokyo 113-8677, Japan; Department of Radiation Oncology, Kurashiki Central Hospital, Okayama 710-8602, Japan; Department of Biostatistics, Yamaguchi University Graduate School of Medicine, Yamaguchi 755-8505, Japan; Department of Radiation Oncology, Graduate School of Biomedical and Health Sciences, Hiroshima University, Hiroshima 734-8553, Japan; Department of Radiation Oncology, Tohoku University Graduate School of Medicine, Miyagi 980-8575, Japan; Department of Radiation Oncology, Kindai University Faculty of Medicine, Osaka 589-8511, Japan; Department of Radiation Oncology, Saitama Cancer Center, Saitama 362-0806, Japan; Department of Radiation Oncology, Kyoto Katsura Hospital, Kyoto 615-8256, Japan; Department of Radiology, Graduate School of Medical Science, University of the Ryukyus, Okinawa 903-0215, Japan; Health Information Management Center, University of the Ryukyus Hospital, Okinawa 903-0215, Japan; Kurume University Radiation Oncology Center, Fukuoka 830-0011, Japan; Division of Radiation Oncology, Department of Radiology, Tokyo Metropolitan Cancer and Infectious Diseases Center Komagome Hospital, Tokyo 113-8677, Japan; Department of Radiation Oncology, Tohoku University Graduate School of Medicine, Miyagi 980-8575, Japan; Department of Radiation Oncology, Graduate School of Medicine, Osaka University, Osaka 565-0871, Japan

**Keywords:** anal canal cancer, chemoradiation, phase II clinical trial, intensity-modulated radiotherapy (IMRT), 3-dimensional conformal radiotherapy (3D-CRT)

## Abstract

This study assessed the efficacy of chemoradiotherapy for squamous cell carcinoma of the anal canal (SCCAC). Patients with T1–4N0-3M0 SCCAC received chemoradiotherapy with 5-fluorouracil (5-FU, 800 mg/m^2^/day, 96-h infusion) and mitomycin-C (MMC, 10 mg/m^2^ bolus). Patients treated with 3-dimensional conformal radiotherapy (3D-CRT) or intensity-modulated radiotherapy (IMRT) were administered 36.0 Gy in 20 fractions or 49.5 Gy in 33 fractions for elective nodal irradiation and 59.4 Gy in 33 fractions for primary tumor and metastatic nodal irradiation. The sample size was considered sufficient to estimate 95% confidence intervals (CIs) for the true 2-year disease-free survival (DFS) within a width of +15% when the expected true 2-year DFS was 70%. The primary endpoint was 2-year DFS. The secondary endpoints were 2-year overall survival (OS), locoregional control (LC), colostomy-free survival (CFS) and adverse events. Thirty-one patients were enrolled between January 2014 and July 2019. The median follow-up was 33.3 months (range, 16.2–65.8 months). Among the 31 patients, 13%, 32%, 16% and 39% had stage I, II, IIIA and IIIB disease, respectively. Thirty patients were treated with IMRT. Complete response (CR) was achieved in 27 patients. The 2-year DFS, OS, LC and CFS rates were 77.4% (95% CI, 58.4–88.5%), 93.5% (95% CI, 76.6–98.3%), 83.9% (95% CI, 65.5–92.9%) and 80.6% (95% CI, 61.9–90.8%), respectively. One patient experienced grade 3 late adverse events; however, no grade ≥ 4 late adverse events occurred. Good DFS with a low rate of late adverse events was observed. Chemoradiotherapy with 5-FU and MMC was effective for SCCAC.

## INTRODUCTION

Squamous cell carcinoma of the anal canal (SCCAC) is a rare disease. SCCAC-related morbidity accounts for 4% of colorectal cancers in the USA, and this number has tripled in the last 30 years [1]. In Japan, the incidence of SCCAC is lower than that in the USA [2]; however, it may yet rise as the increasing incidence of SCCAC is caused by human papillomavirus infection, human immunodeficiency virus infection and older age.

The standard treatment for localized SCCAC remains chemoradiotherapy with 5-fluorouracil (5-FU) and mitomycin-C (MMC), based on the results of several clinical trials in Europe and the USA [3–5]. The RTOG 98–11 trial demonstrated that chemoradiotherapy with 5-FU (1000 mg/m^2^/day, 96-h infusion) and MMC (10 mg/m^2^ bolus) led to significantly better survival, but worse hematotoxicity than that with 5-FU and cisplatin (CDDP) [4, 5]. Prolonged radiotherapy and chemotherapy dose reduction or delay were associated with significantly worse progression-free survival. Furthermore, omission of chemotherapy was associated with worse locoregional control (LC) [6]. In Japan, reduced doses of 5-FU (700–800 mg/m^2^/day, 96-h infusion) and MMC (10 mg/m^2^ bolus) have been used anticipating prolonged radiotherapy or increased toxicity of chemoradiotherapy [7, 8]. In a survey conducted by the Colorectal Cancer Study Group of the Japan Clinical Oncology Group, 25 of 59 patients (45%) were treated with chemoradiotherapy and the complete response (CR) and 3-year progression-free survival rates were 80% and 77%, respectively [9]; these results were similar to those of previous clinical trials in Europe and the USA [3–5]. However, because of the low incidence of SCCAC, few studies in Japan have evaluated chemoradiotherapy with reduced doses of 5-FU and MMC in terms of compliance, efficacy and adverse events. A multicenter prospective study is required to establish the optimal doses of 5-FU, MMC and radiotherapy for Japanese patients with SCCAC.

Therefore, this study aimed to evaluate the efficacy and safety of chemoradiotherapy with reduced dose of 5-FU and MMC in Japanese patients with localized SCCACs.

## PATIENTS AND METHODS

### Patient eligibility

The JROSG 10–2 trial was a multicenter phase II study approved by the Japanese Radiation Oncology Study Group (JROSG) and the institutional review boards of all participating centers (representative institution approval date: September 9, 2013). Written informed consent was obtained from each patient before participating in the study. This study was registered in the Japan Registry of Clinical Trials under number jRCTs041180053.

Patients aged 20–80 years with a histologically confirmed diagnosis of non-metastatic primary SCCAC (cT1–4, any N, M0 according to 7th edition of the UICC TNM classification) were eligible for enrollment. Additional eligibility criteria included a good general condition (Eastern Cooperative Oncology Group performance status 0 or 1) and normal liver, renal and bone marrow function. The exclusion criteria were as follows: (i) a history of malignant disease, (ii) severe heart disease, uncontrolled infection, or metabolic disorders, (iii) any prior pelvic irradiation, (iv) anti-HIV antibody positivity, (v) HBsAg positivity, and (vi) other cases judged inappropriate for this study by the attending doctor.

### Pre-treatment evaluation

Pre-treatment evaluation included the following: general conditions; blood test to determine the adequacy of hepatic, renal and bone marrow function; electrocardiography; chest radiography or chest and abdominopelvic computed tomography (CT) to establish the disease stage; and pelvic magnetic resonance imaging (MRI), colonoscopy and digital rectal examination for evaluation of the primary tumor. Additionally, 2-deoxy-2[F-18]-fluoro-D-glucose positron emission tomography was performed where needed to establish the disease stage. Clinical lymph node metastasis was defined when a lymph node was observed to have a short axis diameter of ≥ 10 mm on CT and MRI images.

### Radiation therapy

Planning CT (axial images at 2–3 mm intervals from the inferior part of the kidney to the femoral shaft) was performed. Patients underwent scanning in the supine position under custom immobilization. Adjusting the bladder volume, such as pooling urine by drinking water, was preferable for reducing the dose to the intestine and fulfilling the bladder dose constraint.

The gross tumor volume (GTV) was contoured using digital rectal examination, imaging and endoscopy and included the primary tumor and metastatic lymph nodes. The clinical target volume (CTV) for the primary tumor was defined as 1.0–2.0 cm expanding to the GTV. No margin for potential microscopic invasion to the metastatic lymph node regions was added to the GTV. The subclinical CTV included the mesorectum, presacrum, bilateral internal and external iliac and bilateral inguinal lymph node regions. The planning target volume (PTV) was set by adding a 5–10-mm margin to the CTV.

A total of 59.4 Gy was delivered with photons (≥6 MV) in 33 fractions for 6.6 weeks (1.8 Gy/day) using 3-dimensional conformal radiation therapy (3D-CRT) or intensity-modulated radiation therapy (IMRT). IMRT with two-step or simultaneous integrated boost (SIB) techniques was acceptable. For patients treated with 3D-CRT or IMRT with the two-step technique, a prescription dose (1.8 Gy/fraction) was administered to the reference point placed at the center of the PTV. Pelvic irradiation was initially administered at 36.0 Gy in 20 fractions, and a further 23.4 Gy was irradiated to the PTV of the primary tumor and metastatic lymph nodes in 13 fractions. For patients treated with IMRT using the two-step technique, 1.8 Gy/fraction was prescribed to D95% for the PTV. If IMRT using the SIB technique was selected, 1.8 Gy/fraction was prescribed to D95% for the PTV for the primary tumor and metastatic lymph nodes and 1.5 Gy/fraction was prescribed for the subclinical PTV minus the primary tumor and metastatic lymph node regions. Table 1 summarizes the dose constraints for the organs at risk. The small bowel was contoured as a peritoneal cavity where it could move. If a dose constraint for the small bowel could not be met, a reduction in the total dose was allowed to meet a dose constraint of the small bowel. Radiotherapy was interrupted in patients with grade ≥ 4 dermatitis and neutropenia.

**Table 1:** Dose constraints for the organs at risk

	**Dose-volume parameter**	**Goal**	**Acceptable**
Small bowel	V30 Gy	< 450 cc	< 500 cc
	V40 Gy	< 200 cc	< 300 cc
	V50 Gy	< 50 cc	< 60 cc
	Dmax	< 55.8 Gy	< 59.4 Gy
Bladder	V35 Gy	< 50%	< 60%
	V40 Gy	< 40%	< 50%
	V50 Gy	< 20%	< 25%
Femoral head	V30 Gy	< 55%	< 60%
	V40 Gy	< 35%	< 40%
	V44 Gy	< 5%	< 10%
Large bowel	V30 Gy	< 200 cc	< 250 cc
	V35 Gy	< 150 cc	< 200 cc
	V45 Gy	< 20 cc	< 50 cc

### Chemotherapy

Two cycles of 5-FU (800 mg/m^2^/day as a 96-h infusion, days 1–4 and 29–32) and MMC (10 mg/m^2^ bolus, maximum 20 mg/body, days 1 and 29) were administered. The patient received appropriate premedication and hydration. The doses of chemotherapy were reduced according to the pre-specified criteria. In cases of grade ≥ 4 neutropenia, the 5-FU and MMC doses were reduced by 50%. The 5-FU dose was reduced by 50% in cases of grade ≥ 3 diarrhea, mucositis and grade 3 dermatitis. Treatment with 5-FU was interrupted in cases of grade ≥ 4 dermatitis.

### Follow-up

Eight weeks after the completion of radiotherapy, digital examination was performed, and the serum squamous cell carcinoma (SCC) antigen level was measured. In addition, abdominopelvic CT, chest CT (or radiography) and colonoscopy were performed 12 weeks after the completion of radiotherapy and repeated every 4 months for 12 weeks to 24 months after the completion of radiotherapy and every 6 months after 24 months.

### Evaluation and response assessment

The primary tumor was confirmed using colonoscopy, and CR for the primary tumor was achieved if the following criteria were met: (i) tumor disappearance, (ii) disappearance of ulcer and erosion, and (iii) no cancer cells found on biopsy. Treatment responses were assessed 12 weeks after the completion of radiotherapy based on the Response Evaluation Criteria in Solid Tumors (RECIST) using CT and/or MRI [10]. Local control was defined as absence of any of the following: (i) endoscopic findings showing that the primary tumor was clearly larger than it was before treatment or that the growth of the tumor exacerbated anal stenosis, and (ii) CT or MRI findings showing that the diameter of tumor increased by ≥ 20% (progressive disease according to the RECIST criteria [8]). Adverse events were graded according to the National Cancer Institute’s Common Terminology Criteria for Adverse Events version 4.0. Adverse events were evaluated weekly during protocol treatment, 8–12 weeks after the completion of radiotherapy, every 4 months for 12 weeks to 24 months and every 6 months after 24 months.

### Statistical analysis

The primary objective of this study was to estimate the 2-year DFS and 95% confidence interval (CI). Thus, 36 patients were required to be enrolled in this study, with planned accrual of 3 years and follow-up of 2 years after the accrual completion. This sample size was considered sufficient to estimate 95% CIs for the true 2-year DFS within a width of +15% when the expected true 2-year DFS was 70%. The secondary endpoints were 2-year overall survival (OS), LC, colostomy-free survival (CFS) and adverse events. Patients who underwent colostomy before treatment were considered colostomy-free if colostomy closure was performed within 1 year after study entry. All the endpoints were measured from the time of study entry to the date of first treatment failure for the given endpoint or to the date of the last follow-up for patients in whom an endpoint did not result in failure. DFS, OS, LC and CFS were estimated using the Kaplan–Meier method.

## RESULTS

### Patient and tumor characteristics

Between January 2014 and July 2019, 31 patients were enrolled in this phase II study at 11 centers in Japan. The number of patients statistically required for this study was 36; however, 31 patients were enrolled as the supply of MMC was suspended from October 2019 onward. The median patient age was 61 years (range, 46–76 years). The clinical tumor stage was cT1 in five patients, cT2 in 16, cT3 in 6 and cT4 in 4. In total, 17 patients presented with clinical evidence of lymph node metastasis (cN+), eight exhibited pararectal lymph node metastasis, three had internal iliac lymph node metastasis, four showed obturator lymph node metastases, and 11 had inguinal lymph node metastasis. Each patient had SCC. Patient and tumor characteristics are summarized in Table 2.

**Table 2:** Patient and tumor characteristics

**Characteristic**	**Value**	
Age	Median 61 years (range; 46–76 years)	
Sex	Male	10 (32%)
	Female	21 (68)
PS	0	25 (81)
	1	6 (19)
Clinical T stage	T1	5 (16)
	T2	16 (52)
	T3	6 (19)
	T4	4 (13)
Clinical N stage	N0	14 (45)
	N1	4 (13)
	N2	8 (26)
	N3	5 (16)
Clinical stage	I	4 (13)
	II	10 (32)
	IIIA	5 (16)
	IIIB	12 (39)
Tumor size	Median 4.0 cm (range; 1.5–10.0 cm)	
Colostomy at pre-treatment	Yes	3 (10)
	No	28 (90)
SCC value before treatment	Median 1.8 ng/ml, (range; 0.4–36.3)	

Abbreviations: PS, performance status; SCC, squamous cell carcinoma antigen

### Tolerability

All patients received a planned dose of radiotherapy and two cycles of 5-FU and MMC. IMRT was performed in 13 patients using the two-step technique, IMRT with SIB in 16, 3D-CRT in 1 and IMRT for pelvic irradiation plus a 3D-CRT boost in 1. The median total dose was 59.4 Gy (range, 55.8–59.4 Gy). Two and four patients received 57.6 Gy and 55.8 Gy, respectively, according to the dose constraints of the small intestine. Radiotherapy was interrupted in four patients because of grade 4 hematologic toxicity, and the median interruption period of the radiotherapy was 2.5 days (range, 1–6 days). The median duration of radiation treatment was 47 days (range, 43–56 days). Administration of the second cycle of chemotherapy was delayed in five patients. Five patients received reduced doses of the second cycle 5-FU and MMC, and one patient received a reduced dose of 5-FU for grade ≥ 4 hematologic toxicity, grade ≥ 3 diarrhea and severe weight loss.

### Safety

Grade 4 acute hematologic toxicity, which included neutropenia (12.9%) and thrombocytopenia (9.7%), was observed in seven patients (22.6%). However, acute grade ≥ 4 non-hematologic toxicities were not observed. The most common types of acute grade 3 adverse events were hematologic, radiation dermatitis, radiation mucositis, diarrhea and pain events (Table 3).

**Table 3:** Acute adverse events (*n* = 31)

		**Grade**		
	**1**	**2**	**3**	**4**
Hematologic				
Leucopenia	4 (13%)	10 (32)	15 (48)	0 (0)
Neutropenia	7 (23)	8 (26)	10 (32)	4 (13)
Febrile neutropenia	0 (0)	0 (0)	0 (0)	0 (0)
Anemia	14 (45)	12 (39)	1 (3)	0 (0)
Thrombocytopenia	10 (32)	5 (16)	5 (16)	3 (10)
T-Bil	1 (3)	0 (0)	0 (0)	0 (0)
AST	9 (29)	4 (13)	0 (0)	0 (0)
ALT	9 (29)	2 (7)	2 (7)	0 (0)
Creatinine	4 (13)	1 (3)	0 (0)	0 (0)
Non-hematologic				
Fatigue	8 (26)	3 (10)	1 (3)	0 (0)
Anorexia	11 (35)	6 (20)	1 (3)	0 (0)
Nausea	8 (26)	5 (16)	2 (7)	0 (0)
Diarrhea	6 (19)	6 (19)	4 (13)	0 (0)
Constipation	4 (13)	1 (3)	0 (0)	0 (0)
Radiation dermatitis	6 (19)	12 (39)	10 (32)	0 (0)
Radiation mucositis	5 (16)	7 (23)	9 (29)	0 (0)
Anal pain	4 (13)	15 (48)	4 (13)	0 (0)
Anal hemorrhage	14 (45)	2 (7)	0 (0)	0 (0)
Urinary frequency	5 (16)	0 (0)	0 (0)	0 (0)
Urinary retention	0 (0)	0 (0)	0 (0)	0 (0)

Abbreviations: T-Bil, total bilirubin; AST, aspartate aminotransferase; ALT, alanine aminotransferase

No grade 4 late adverse events were observed at the time of analysis. Only one patient (3.2%) experienced grade 3 pelvic bone fracture. Grade 1 radiation proctitis and grade 2 anal bleeding were observed in three (9.7%) and two patients (6.5%), respectively; however, grade ≥ 3 bleeding was not. All grades of late toxicity are shown in Table 4.

**Table 4:** Late adverse events

	**Grade (N = 31)**			
	**1**	**2**	**3**	**4**
Pelvic bone fracture	0 (0%)	1 (3.2)	1 (3.2)	0 (0)
Fecal incontinence	1 (3.2)	0 (0)	0 (0)	0 (0)
Radiation proctitis	3 (9.7)	0 (0)	0 (0)	0 (0)
Anal bleeding	0 (0)	2 (6.5)	0 (0)	0 (0)
Vaginal obstruction	0 (0)	1 (3.2)	0 (0)	0 (0)
Anal pain	1 (3.2)	1 (3.2)	0 (0)	0 (0)
Perineal pain	1 (3.2)	0 (0)	0 (0)	0 (0)
Frequent defection	1 (3.2)	0 (0)	0 (0)	0 (0)
Constipation	0 (0)	1 (3.2)	0 (0)	0 (0)
Radiation colitis	2 (6.5)	0 (0)	0 (0)	0 (0)
Ileus	0 (0)	1 (3.2)	0 (0)	0 (0)

### Survival and relapse

**Fig. 1:**
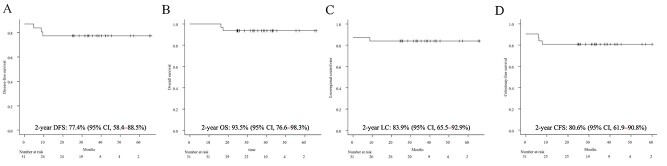
Disease-free survival (DFS) (A), overall survival (OS) (B), local control (LC) (C) and colostomy-free survival (CFS) (D).

The median follow-up period was 33.3 months (range, 16.2–65.8 months). A total of 27 patients achieved CR. At the time of analysis, local recurrence was observed in five patients, and four of the five patients who could not achieve CR experienced residual primary tumor progression. Pelvic lymph node recurrence was also observed in one patient. Distant metastasis was observed in three patients (in the liver in two patients and in the para-aortic lymph node in one). Consequently, three of the 29 survivors were alive with cancer and two died because of SCCAC at the time of analysis. The 2-year DFS, OS and LC rates were 77.4% (95% CI, 58.4–88.5%), 93.5% (95% CI, 76.6–98.3%) and 83.9% (95% CI, 65.5–92.9%), respectively (Fig. 1A–C). Colostomy was performed in five patients at the time of analysis. One of the three patients who underwent pre-treatment colostomy underwent colostomy closure 17.5 months after registration, and colostomy was performed in three patients owing to residual tumor after chemoradiotherapy. However, none of the patients who received LC underwent colostomy because of fecal incontinence induced by radiotherapy. The 2-year CFS rate was 80.6% (95% CI, 61.9–90.8%) (Fig. 1D).

## DISCUSSION

In the current study, all the patients received two cycles of 5-FU, and MMC and completed the planned radiotherapy dose. The 2-year DFS was 77.4% (95% CI, 58.4–88.5%), which was similar to that reported in previous studies evaluating the efficacy of chemoradiotherapy with 5-FU and MMC for patients with SCCAC (Table 5) [4, 5, 11–14]. The 2-year OS of the current study (93.5%; 95% CI, 76.6–98.3%) was excellent compared to that reported in previous studies (65–78% of the 3- or 5-year OS), wherein the patients were treated with 3D-CRT [3–5, 11, 15], and was similar (86% of the 2-year OS) to that reported in the RTOG 0529 trial, which included patients treated with IMRT [15, 16]. Elson *et al.* reported that in a National Cancer Database study, patients with SCCAC treated with IMRT were at a reduced risk of a long treatment time compared to those treated with 3D-CRT, and the 5-year OS in patients treated with IMRT (80.8%) was significantly higher than that of patients treated with 3D-CRT (78.9%) (*P* = 0.0036) [16]. In the current study, 30 of 31 patients (97%) received pelvic irradiation with IMRT, and only four patients (13%) underwent interrupted radiotherapy during a short period (median, 2.5 days). This might have caused the excellent OS.

**Table 5:** List of characteristics in previous and current studies

Reference **(ye**ar)	**Study type**	**Treatment**	**Number of patients**	**Stage I/II/III/IVA (n)**	**Radiotherapy; total dose**	**Chemotherapy; total dose**	**OS**	**DFS**	**LC**	**CFS**
[3] (1996)	Phase 3	RT alone	278	-	45 Gy plus 15–20 Gy boost* or surgery	-	58.0% (3 yr)	-	39.0% (3 yr)	-
		CRT (5-FU + MMC)	283	-	45 Gy plus 15–20 Gy boost* or surgery	5-FU; 100 mg/m^2^/day, days 1–4, or 750 mg/m^2^/day, days 1–5 MMC; 12 mg/m^2^, day1	65.0	-	61.0	-
[4, 5] (2008, 2012)	Phase 3	CRT (5-FU + MMC)	324	150/62/33/67	55–59 Gy	5-FU; 1000 mg/m^2^/day, days 1–4, 29–32 MMC; 10 mg/m^2^, days 1, 29	78.3 (5 yr)	67.8 (5 yr)	80.0 (5 yr)	71.9 (5 yr)
		CRT (5-FU + CDDP) + adjuvant 5-FU + CDDP	320	152/53/24/76	55–59 Gy	5-FU; 1000 mg/m^2^/day, days 1–4, 29–32, 57–60, 85–88 CDDP; 75 mg/m^2^, days 1, 29, 32, 57, 85	70.7	57.8	73.6	65.0
[13, 14] (2013, 2022)	Phase 2	CRT (5-FU + MMC)	52	I + II/III/IVA 24/28/0	50.4–54.0 Gy	5-FU; 1000 mg/m^2^/day, days 1–4, 29–32 MMC; 10 mg/m^2^, days 1, 29	86 (2 yr)	76 (2 yr)	86 (2 yr)	84 (2 yr)
Current study	Phase 2	CRT (5-FU + MMC)	31	4/10/17/0	59.4 Gy	5-FU; 800 mg/m^2^/day, days 1–4, 29–32 MMC; 10 mg/m^2^, days 1, 29	93.5 (2 yr)	77.4 (2 yr)	83.9 (2 yr)	80.6 (2 yr)

Abbreviations: OS, overall survival; DFS, disease free survival; LC, locoregional control; CFS, colostomy-free survival; RT, radiotherapy; CRT, chemoradiotherapy; 5-FU, 5-fluorouracil; MMC, mitomycin-C; yr, year

^*^Additional 15–20 Gy boost was delivered when > 50% response was observed 6 weeks after the completion of 45 Gy.

In the current study, local recurrence at the time of analysis was observed in five patients, and four of the five patients who could not achieve CR experienced residual primary tumor progression. The 2-year LC rate was 83.9%. These results were similar to those of previous studies [4, 5, 13, 14]. CFS is mainly a measure of anal sphincter preservation after chemoradiotherapy, and in previous studies, the 2–5-year CFS was 73–88%, similar to that in this study (2-year CFS: 80.6%; 95% CI, 61.9–90.8%) (Table 5) [4, 5, 11, 13–15]. Colostomy is generally performed after chemoradiotherapy because of failure to control anal canal cancer and/or chemoradiotherapy-related complications. Up to 15–24% of colostomies were performed owing to treatment [3, 17, 18], and Sunesen *et al.* reported that 83% of treatment-related colostomies were performed within 2 years after the completion of radiotherapy [17]. In the current study, no patient underwent colostomy owing to treatment at the time of analysis (median follow-up periods: 33.3 months).

Acute hematologic toxicity caused by chemoradiotherapy with 5-FU and MMC was significantly higher than that caused by 5-FU and CDDP in the RTOG 98–11 trial (61% vs 42%, *P* < 0.001) [4, 5]. The rate of acute hematologic grade 3 or 4 toxicity in the current study was 62%, similar to that observed in previous studies [4, 5, 13, 14]. Recent reports showed that bone marrow-sparing IMRT could reduce grade ≥ 3 acute hematologic toxicity to 19–28% in patients with SCCAC receiving chemoradiotherapy with 5-FU and MMC [19, 20]. Because dose constraints for the bone marrow were not set in the current study, the rate of grade ≥ 3 acute hematologic toxicity was not lower than that reported in previous studies in which 3D-CRT was used. The proportion of cases of grade ≥ 3 acute gastrointestinal toxicity and dermatitis in the current study (16% and 32%, respectively) were similar to that in the RTOG 0529 with IMRT (21% and 23%, respectively) [13, 14] and lower than that in the RTOG 9811 with 3D-CRT (36% and 49%, respectively) [4, 5]. The rate of acute non-hematologic toxicity might be similar to that of IMRT because almost all patients in the current study (97%) were treated with IMRT. Grade ≥ 3 late toxicity was only observed in one patient (grade 3 pelvic bone fracture) in the current study, and this result was lower than that reported in previous studies (13–20%) [4, 5, 13, 14]. Of the 31 patients in the current study, 25 (81%) received a total dose of 59.4 Gy; however, no patient experienced grade ≥ 3 radiation proctitis or anal bleeding, and colostomy was performed owing to treatment toxicity.

The optimal dose of chemoradiotherapy for SCCAC remains controversial. In the UKCCCR and ACCORD03 trials, a total dose of ≥60 Gy was administered with center split [3, 11], and selective doses in previous studies ranged from 50.4 to 59.4 Gy. These were administered according to the T stage and size of lymph node metastasis [4, 5, 13–15]. Shah *et al.* investigated the optimal dose of chemoradiotherapy for SCCAC using the National Cancer Database and reported that 45–54 Gy was associated with worse survival than 54 Gy (*P* = 0.009); however, in cases of locally advanced disease, survival did not differ significantly between 54 Gy and > 54–60 Gy (*P* = 0.166) [21]. Moreover, the ACT5 trial of PLATO (ISRCTN88455282) included patients with locally advanced high-risk anal cancer and investigated the role of CRT with dose escalation up to 61.6 Gy in 28 fractions. The Japan Clinical Oncology Group conducted a phase I/II trial of chemoradiation with S-1, MMC, and a total dose of 59.4 Gy for patients with clinical stage II/III SCCAC [22]. Therefore, evaluating the efficacy of chemoradiotherapy (59.4 Gy) was important in the current study.

In Japan, a reduced dose of 5-FU (700–800 mg/m^2^/day) has been used considering the increased toxicity of chemotherapy [7, 8]. The current study set a total dose of 59.4 Gy and a reduced 5-FU dose of 800 mg/m^2^/day and reported similar results, including those for efficacy and toxicity, to those reported in North American and European studies that used a standard dose of 5-FU (1000 mg/m^2^/day) and a total dose of 50.4–59.4 Gy [3–5, 13, 14]. Previous Japanese reports showed that excellent 5-year OS and LC rates (100% and 92%, respectively) were obtained for 13 patients with SCCAC treated with chemoradiotherapy or radiotherapy alone (median total dose; 59.4 Gy) [7]. Ten of 13 patients received chemoradiotherapy with 5-FU (800 mg/m^2^/day) and MMC (10 mg/m^2^). No interruption of radiotherapy was needed for any patient, and only one patient experienced a grade 3 late adverse event (femoral head necrosis). Compared to the current study, higher OS and LC rates were obtained, possibly owing to the inclusion of a higher population (38%) of patients with stage I SCCAC (10% in the current study).

The number of patients statistically required for this study was 36; however, only 31 patients were enrolled as the supply of MMC was suspended from October 2019 onward. Therefore, we could not conclude whether the results of this study were positive or negative. Furthermore, various radiotherapy techniques such as 3DCRT, IMRT with the two-step technique, and IMRT with SIB were included, and biomarkers such as human papilloma virus and p16 were not routinely screened. Further studies are needed to establish the optimal dose of radiotherapy according to risk factors, such as T/N stage and biomarker levels and an optimal radiotherapy technique, including dose constraints for organs at risk.

The required number of eligible patients was not reached in the current study. However, this multicenter phase II study showed excellent compliance and demonstrated that chemoradiotherapy with 59.4 Gy plus a reduced dose of 5-FU (800 mg/m^2^/day) and MMC for SCCAC might lead to good DFS (77.4%, 95% CI, 58.4–88.5%) at 2 years, with a low rate of late adverse events. IMRT is recommended for definitive chemoradiotherapy in patients with SCCAC.

In conclusion, good DFS with a low rate of late adverse events was observed. Chemoradiotherapy with 5-FU and MMC and administration of a total dose of 59.4 Gy for patients with anal canal cancer was an effective definitive treatment.

## CLINICAL TRAIL REGISTRATION NUMBER

This study was registered in the Japan Registry of Clinical Trials under number jRCTs041180053.

## CONFLICT OF INTEREST

The author has no conflicts of interest to disclose.
